# The impact of age, gender, and ocular parameters on corneal biomechanics in Chinese refractive candidates

**DOI:** 10.1371/journal.pone.0325419

**Published:** 2025-06-04

**Authors:** Jiliang Ning, Siyu Sun, Taorui Yu, Xiaoyu Liu, Lin Jin, Lijun Zhang

**Affiliations:** 1 Department of Ophthalmology, The Third People’s Hospital of Dalian, Dalian, China; 2 Department of Ophthalmology, Dalian Municipal Eye Hospital, Dalian, China; 3 Liaoning Provincial Key Laboratory of Cornea and Ocular Surface Diseases, Dalian, China; 4 Liaoning Provincial Optometry Technology Engineering Research Center, Dalian, China; Aravind Eye Hospital, INDIA

## Abstract

We aimed to quantify the distribution of corneal biomechanics in healthy, myopic Chinese candidates for refractive surgery using an ocular response analyzer, and explore factors that influence corneal biomechanics. In this retrospective cross-sectional study, 1,090 eyes from 1090 myopic patients scheduled for refractive surgery at the Refractive Center of Dalian Third People’s Hospital between January 2021 and May 2022 were examined. Participants were categorized into three groups based on spherical equivalent power: low myopia (SE> −3.00D), moderate myopia (>−6.00D < SE ≤ −3.00D), and high myopia (SE ≤ −6.00D); then further categorized by central corneal thickness into four groups: < 500μm, 500 to <550μm, 550 to <600μm, and ≥600μm. The Ocular Response Analyzer was used to measure corneal hysteresis (CH) and corneal resistance factor (CRF). Differences in CH and CRF were compared across groups. Generalized estimating equations were applied to determine correlations between CH and CRF and variables such as sex, age, corneal keratometry, central corneal thickness, white-to-white corneal diameter, and spherical equivalent power. The average corneal hysteresis (CH) was 11.18 ± 1.35 mmHg, while the average corneal resistance factor (CRF) was 11.22 ± 1.64 mmHg. The average CRF values were significantly higher in men than in women (P < 0.05). In the high myopia group, CH values were significantly lower than those in the moderate and low myopia groups (P < 0.01). CH and CRF increased with corneal thickness across all groups, with statistically significant differences (P < 0.01). Generalized estimating equations showed a positive correlation between corneal thickness and curvature with both CH and CRF (all P < 0.01). Age was negative correlation between CH and CRF (all P < 0.05). Spherical equivalent power was positively correlated with CH (P < 0.01). Corneal thickness, curvature, and refractive power significantly affected corneal measurement values in individuals with myopia. The CH and CRF values increased with increasing corneal thickness and curvature, and CH was negatively correlated with the severity of myopia.

## Introduction

The cornea is a crucial component of the eye’s refractive system, contributing approximately 70% of its refractive power. Its viscoelastic biomechanical properties enable it to maintain optimal refractive and mechanical characteristics in response to various internal and external pressures, demonstrating a balance between softness and toughness [1]. Measuring corneal biomechanics is vital for diagnosing and prognosing various diseases, including screening for corneal ectasia and predicting various outcomes following corneal refractive surgery [2,3]. The ocular response analyzer (ORA; Reichert Ophthalmic Instruments, Buffalo, New York, USA) is widely used to measure corneal biomechanics in vivo [4]. It uses infrared signals and variable pulse airflow to assess corneal pressure changes during bidirectional applanation, providing two key parameters: corneal hysteresis (CH) and corneal resistance factor (CRF). CH represents the pressure difference between the two corneal applanations, indicating energy dissipation of the cornea during the stress process [5]. In contrast, the CRF is more closely related to the pressure experienced during the first applanation event and resistance to the initial elastic deformation [5]. Studies have explored corneal biomechanics across various ethnic groups [3,6–9]. However, there is currently a lack of large-sample studies investigating reference values for corneal viscoelastic properties in healthy Chinese candidates for myopic refractive surgery. The aim of this study was to establish reference values for corneal biomechanical measurements in this population. Additionally, we seek to investigate the effects of sex, age, corneal keratometry (K), central corneal thickness (CCT), white-to-white (WTW) corneal diameter, and spherical equivalents (SE) on corneal biomechanics.

## Materials and methods

### Research subjects

In this retrospective cross-sectional analysis, we included 1090 eyes of 1,090 patients with myopia (random selection of one eye of each patient) scheduled to undergo refractive surgery at the Refractive Center of the Dalian Third People’s Hospital between January 2021 and May 2022. Data were accessed for research purposes starting on February 3, 2023. The cohort consisted of 578 males and 512 females, aged 16–40 years, with an average age of 23.49 ± 6.42 years. Inclusion criteria required participants to have an annual progression of myopia not exceeding 0.50 D, no contact lens use or cessation of soft contact lenses for at least two weeks, and discontinuation of rigid lenses for more than three weeks or orthokeratology lenses for three months. Patients with keratoconus, suspected keratoconus, corneal dystrophy, glaucoma, diabetes, scarring, systemic diseases, previous eye surgeries, or eye trauma were excluded. The research protocol received approval from the Ethics Committee of the Dalian Third People’s Hospital (Approval Number: 2022-039-001). This study adhered to the principles outlined in the Declaration of Helsinki. In accordance with the requirements set forth by the ethics committee, the waiver of patient informed consent was granted. The authors did not have access to any information that could identify individual participants during or after the data collection process.

### Method of examination

ORA was used to assess central CH and CRF in the examined eyes. During measurements, patients were instructed to relax and focus on a green light to maintain target fixation. Once the pressure sensor was properly aligned with the cornea, a gentle pulse of air was emitted, interacting with the cornea. After 3 milliseconds, the airflow stopped, and the device recorded a waveform and corresponding values. Each eye was measured three times, and the result with a signal score above 6 was included in the analysis. The Pentacam (Oculus Inc., Wetzlar, Germany) was used to measure the anterior segment parameters, including corneal keratometry (K), central corneal thickness (CCT), and corneal white-to-white (WTW) diameter. Spherical equivalent (SE) was determined from cycloplegic computer refraction results. All tests were performed using the same equipment by an experienced technician.

### Statistical analysis

Statistical analyses were performed using SPSS version 26.0 (SPSS, Chicago, Illinois, USA). The normality of the measurement indicators in this study was assessed using the Kolmogorov-Smirnov test, which confirmed a normal distribution. Data are expressed as mean ± standard deviation (SD) with 95% confidence intervals (CI). An independent samples t-test was employed to compare biomechanical parameters between sexes. One-way analysis of variance (ANOVA) was used to evaluate the overall differences in corneal biomechanical parameters across various spherical equivalent (SE) and central corneal thickness (CCT) groups, with post-hoc comparisons performed using the Bonferroni test. Generalized estimating equations were utilized to examine the correlations between CH, CRF, sex, age, CCT, corneal keratometry (K), white-to-white (WTW) corneal diameter, and SE. Statistical significance was set at p < 0.05.

## Results

This study involved 1,090 participants (578 males and 512 females) with an average age of 23.49 ± 6.42 years. The average SE, CCT, K, and corneal WTW diameter of the participants were −4.53 ± 2.12 D, 552.34 ± 30.16 μm, 43.31 ± 1.38 D, and 11.80 ± 0.41 mm, respectively. The mean CH and CRF were 11.18 ± 1.35 mmHg and 11.22 ± 1.64 mmHg, respectively. The results are summarized in Table 1.

**Table 1:** Demographic data, ocular parameters, and corneal biomechanical parameters of the subjects.

Parameter	Mean± SD	95% CI	Range
Number of eyes	1090		
Sex (male/female)	578/512		
Age, years	23.49 ± 6.42	23.11–23.87	16–40
SE, D	−4.53 ± 2.12	−4.66 to −4.41	−14.75 to 0
CCT, μm	552.34 ± 30.16	550.54–554.13	467–643
Corneal keratometry, D	43.31 ± 1.38	43.23–43.39	37.96–47.15
WTW, mm	11.80 ± 0.41	11.78–11.82	10.6–13.1
IOP, mmHg	15.56 ± 2.73	15.40–15.72	8.5–25
CH, mmHg	11.18 ± 1.35	11.10–11.26	6.2–17.4
CRF, mmHg	11.22 ± 1.64	11.12–11.32	6.2–17.3

SE, spherical equivalent; CCT, central corneal thickness; WTW, white-to-white corneal diameter; IOP, intraocular pressure;CH, corneal hysteresis; CRF, corneal resistance factor.

Table 2 presents the comparison of corneal biomechanics between sexes. The mean CH value for males was 11.23 ± 1.37 mmHg, and 11.13 ± 1.32 mmHg for females, with no statistically significant difference (P = 0.246). However, the mean CRF value for males (11.32 ± 1.67 mmHg) was significantly higher than that for females (11.11 ± 1.60 mmHg), with a statistically significant difference (P < 0.05).

**Table 2:** Sex differences in corneal biomechanics among healthy individuals.

	Male(Mean±SD)	Female(Mean±SD)	t	P
n	578	512		
CH, mmHg	11.23 ± 1.37	11.13 ± 1.32	1.160	0.246
CRF, mmHg	11.32 ± 1.67	11.11 ± 1.60	2.094	0.036

CH, corneal hysteresis; CRF, corneal resistance factor.

Table 3 shows the differences in corneal biomechanics across varying diopters. As the diopters increased, CH value decreased. The Dunn-Bonferroni post hoc test showed no statistical difference between the low and moderate myopia groups (P = 0.358), but all other comparisons were significant (P < 0.01). In contrast, no significant difference was identified in CRF across the diopter groups (P = 0.846).

**Table 3:** Diopter differences in corneal biomechanics among healthy individuals.

	Low myopia (Mean±SD)	Moderate myopia (Mean±SD)	High myopia (Mean±SD)	F	P
n	229	609	252		
CH, mmHg	11.38 ± 1.29	11.21 ± 1.31	10.94 ± 1.46	6.774	0.001
CRF, mmHg	11.21 ± 1.64	11.25 ± 1.65	11.18 ± 1.64	0.167	0.846

CH, corneal hysteresis; CRF, corneal resistance factor.

Table 4 compares corneal biomechanics across varying corneal thicknesses. The examined eyes were grouped into four categories: < 500 μm, 500 to <550 μm, 550 to <600 μm, and ≥600 μm. The average CH values for these four groups were 9.90 ± 1.08, 10.69 ± 1.16, 11.53 ± 1.23, and 12.45 ± 1.48 mmHg, respectively, while the average CRF values were 9.38 ± 1.15, 10.54 ± 1.40, 11.67 ± 1.39, and 13.17 ± 1.74 mmHg, respectively. Both CH and CRF values increased sequentially with corneal thickness, and the overall difference being statistically significant (P < 0.01). The Dunn-Bonferroni post hoc test confirmed that the differences between the subgroups were statistically significant (P < 0.01).

**Table 4:** Corneal thickness in corneal biomechanics among healthy individuals.

CCT, μm	<500	500-550	550-600	>600	F	P
n	43	454	512	81		
CH, mmHg	9.90 ± 1.08	10.69 ± 1.16	11.53 ± 1.23	12.45 ± 1.48	83.053	0.000
CRF, mmHg	9.38 ± 1.15	10.54 ± 1.40	11.67 ± 1.39	13.17 ± 1.74	127.939	0.000

CCT,central corneal thickness; CH, corneal hysteresis; CRF, corneal resistance factor.

Generalized estimating equations were used to assess correlations between CH, CRF, sex, age, CCT, K, WTW corneal diameter, and SE (Table 5). Age, CCT, K, and SE values were correlated with CH. Specifically, SE, CCT, K, and CH exhibited positive correlations (all P < 0.01), whereas age and CH showed a negative correlation (P < 0.05). CCT, K, and CRF were positively correlated (all P-values < 0.01). In contrast, age, WTW, and CRF demonstrated a negative correlation (P < 0.05).

**Table 5:** Analysis of influencing factors of corneal biomechanics.

Parameter		CH	CRF
Sex	*β*	−0.028	0.058
	*SE*	0.084	0.093
	*P*	0.737	0.533
Age, year	*β*	−0.012	−0.021
	*SE*	0.006	0.007
	*P*	0.040	0.001
CCT,μm	*β*	0.022	0.031
	*SE*	0.001	0.001
	*P*	0.000	0.000
Corneal keratometry,D	*β*	0.125	0.106
	*SE*	0.029	0.034
	*P*	0.000	0.002
WTW,mm	*β*	−0.182	−0.256
	*SE*	0.099	0.116
	*P*	0.065	0.028
SE, D	*β*	0.073	0.011
	*SE*	0.019	0.022
	*P*	0.000	0.613

CCT, central corneal thickness; WTW, white-to-white corneal diameter; |SE|, absolute value of equivalent sphericity; *β*, unstandardized beta co-efficient; *SE*, standard error.

## Discussion

In recent years, advancements in corneal refractive surgery technology have improved outcomes for myopia correction. However, post-refractive ectasia can still occur in eyes without identifiable preoperative risk factors, with an incidence ranging from 0.011% to 0.09% [10]. Biomechanical decompensation plays a key role in corneal dilatation after refractive surgery [11], making it crucial to understand the biomechanical properties of myopic eyes to assess the predictability and safety of these surgeries. Our study found that the average CH and CRF values were 11.19 ± 1.37 mmHg and 11.22 ± 1.67 mmHg, respectively. Previous studies have reported CH and CRF distributions in healthy individuals. In a study of 2,039 healthy Caucasians, the average CH and CRF were 11.49 ± 1.89 mmHg and 11.40 ± 2.30 mmHg, respectively [3]. Among healthy Japanese participants, the average CH was 10.2 ± 1.3 mmHg [12], while healthy Thais had an average CH of 10.18 ± 1.48 mmHg. In healthy Saudi adults, CH averaged 11.16 ± 2.11 mmHg, and CRF averaged 11.07 ± 2.31 mmHg [6,8]. Differences between our study and these studies may be attributed to racial and geographic variations. Establishing population-specific norms for corneal biomechanics is important. This study, the largest of its kind, offers valuable insights for the preoperative evaluation of corneal refractive surgery candidates using the ORA.

Gender is generally not considered to significantly influence corneal biomechanics. In this study, we compared CH and CRF values in myopia patients by gender and found no significant difference in CH values between male and female patients. However, CRF was higher in males. After adjusting for confounding factors such as age, corneal thickness, corneal curvature, and diopter using generalized estimating equations, no statistically significant differences in CH and CRF were found between sexes. This aligns with previous studies by Oncel and El Massry et al., which reported no significant influence of gender on corneal biomechanical properties in myopic eyes [13,14]. Bahadir et al. measured the CH and CRF levels at various time points during the menstrual cycle (before, during, and after ovulation) in young women [15]. They found no significant changes in corneal biomechanics at different points in the measurement cycle, suggesting that estrogen and progesterone levels do not affect corneal biomechanics in non-pregnant women.

When myopic patients were categorized according to spherical equivalents (SE), CRF values did not differ significantly across myopia levels, but CH decreased as myopia increased. Our analysis indicated a relationship between CH and SE; however, we did not find any notable correlation between CRF and SE. This is consistent with earlier studies, which also noted a decrease in CH with increasing myopia [16–19]. In a similar vein, research conducted by Song and colleagues, along with Bueno-Gimeno and their team, identified an inverse relationship between axial length and CH [20,21]. Increasing myopia is associated with thinner scleral collagen fibers, decreased collagen content, and relatively low proteoglycan synthesis [22–24], contributing to scleral thinning and diminished mechanical properties. Given that the corneal stroma is a continuation of the sclera, changes in the sclera’s structure may reduce corneal viscoelasticity. This raised concerns about postoperative biomechanical stability in highly myopic patients undergoing corneal refractive surgery.

Corneal thickness is another significant factor affecting corneal viscoelasticity. In this study, CH and CRF values increased with greater corneal thickness. After controlling for sex, age, and other ocular parameters, we found a positive correlation between CH, CRF, and corneal thickness. These findings align with those of previous research [25–27]. Fontes et al. reported that even when corneal thickness was equalized, patients with keratoconus had lower CH and CRF values than those with healthy thin corneas [28]. Galletti also noted that adjusting for corneal thickness improved the detection of keratoconus using the corrected CRF index [29]. Establishing standardized ranges for CH and CRF across varying corneal thickness is important for assessing the normality of corneal biomechanics.

Age-related changes in corneal structure, including the growth of stromal fibrils and increased cross-linking [30], can also affect corneal biomechanics. In vitro experiments have shown that corneal stiffness, as measured by Young’s modulus, increases with age, while the hysteresis area decreases [31]. In vivo studies have confirmed a significant association among age, CH, and CRF. Ahmed et al. in a study of 997 eyes from 508 individuals aged 10–84 years, reported a considerable inverse correlation between age and both CH and CRF [14]. Similarly, Kamiya et al. found an inverse correlation between age and CH in a study of 204 healthy individuals aged 19–89 years [32]. Consistent with the aforementioned studies, our analysis model demonstrates that age has a statistically significant correlation with both CH and CRF. Our study also shows a positive correlation between corneal curvature and both CH and CRF, indicating that a steeper cornea has a stronger resistance to pressure, which aligns with the findings of Hwang et al [33]. Francis et al. and Matsumoto also suggested that corneal curvature influences corneal stiffness, with a steeper cornea corresponding to increased hardness [34,35]. This may be due to the fact that a steeper cornea requires greater deformation to achieve flattening, necessitating increased pressure, which in turn affects ORA measurement.

This study had several limitations. First, the participants were predominantly young, which may limit the generalizability of the findings. Future studies ought to encompass a wider age spectrum to delve deeper into how aging influences corneal biomechanics. Second, cross-sectional designs may hinder the ability to establish causal relationships; therefore, longitudinal studies with larger cohorts may be necessary to validate these findings in the future. Third, while participants under 18 years of age may lack refractive stability. China’s clinical guidelines (as outlined in ophthalmic expert consensus documents) permit corneal refractive surgery (e.g., LASIK, SMILE) for minors with myopia who demonstrate compelling career-specific requirements (such as eligibility criteria for military or police academy enrollment). This exception is strictly conditional upon comprehensive preoperative evaluation and documented informed consent obtained from both the minor and their legal guardian(s).

In summary, this study outlines the biomechanical characteristics of Chinese refractive surgery candidates and provides reference values for CH and CRF across different refractive powers and corneal thicknesses. Additionally, we examined the correlations between CH, CRF, and factors such as sex, age, corneal thickness, diopters, corneal keratometry, and corneal diameters. Our results show that corneal thickness and curvature are positively correlated with CH and CRF, while age and higher myopia are negatively correlated with CH.

## Supporting information

S1 Dataset Dataset for 1090 patients. (XLSX)
